# Investigating the safety of antibiotics added to collared peccary (*Pecari tajacu*) semen extender through a multiparametric thermoresistance test

**DOI:** 10.1590/1984-3143-AR2024-0018

**Published:** 2024-08-05

**Authors:** Caio Sérgio Santos, Yasmim Carla da Silva Cavalcante, Lívia Batista Campos, Andréia Maria da Silva, Francisco Marlon Carneiro Feijó, Alexandre Rodrigues Silva

**Affiliations:** 1 Laboratório de Conservação de Germoplasma Animal, Departamento de Ciências Animais, Universidade Federal Rural do Semi-Árido – UFERSA, Mossoró, RN, Brasil; 2 Laboratório de Microbiologia e Virologia, Departamento de Ciências Animais, Universidade Federal Rural do Semi-Árido – UFERSA, Mossoró RN, Brasil

**Keywords:** antibacterial, extender, sperm, thermoresistance, toxicity

## Abstract

The effects of antibiotics on sperm longevity in collared peccary (*Pecari tajacu*) fresh diluted semen was evaluated. Semen samples from six adult males were collected by electroejaculation and diluted in Tris-citrate-fructose alone (control) and plus streptomycin-penicillin (2 mg/ml-2000 IU/ml) or gentamicin (70 µg/ml). Membrane integrity and functionality, mitochondrial activity and sperm morphology were assessed subjectively. Sperm motility and other kinetic parameters were objectively assessed using CASA (computer-assisted semen analysis). The semen diluted according to the treatments were submitted to the thermoresistance test, incubated at 37 ° C, and the sperm parameters analyzed at 0, 30, 60, 120 and 180 min. The average values of the treatments were compared with each other and between the times. There were no differences (P > 0.05) between treatments until the end of the test. Control and streptomycin-penicillin samples maintained sperm function for up to 180 min (with total motility of 24.3 ± 7.1% and 28 ± 8.7%, respectively). Gentamicin aliquots retained most parameters until the end of the incubation, except for membrane integrity and mitochondrial activity that declined (P < 0.05) at 180 min (53.1 ± 7.1% and 50.7 ± 6.2%, respectively) compared to 0 min (80.5 ± 4.7% and 86.3 ± 3.4%, respectively). In conclusion, a multiparametric thermoresistance test proved that Tris-based extenders used for collared peccary semen can be effectively supplemented by streptomycin-penicillin (2 mg/ml-2000 IU/ml) or gentamicin (70 µg/ml), especially during 180-min incubation at 37 °C.

## Introduction

The collared peccary (*Pecari tajacu*) is a wild species native to the American continent that has great ecological importance in the ecosystems it inhabits. In terms of conservation, its global population has been classified as Least Concern (Gongora et al., 2011); however, the number of individuals has been drastically reduced in some biomes because of anthropic interference through poaching and defragmentation of their habitat (Desbiez et al., 2012). Thus, several studies have been conducted to develop assisted reproduction protocols for its conservation, mainly those related to refrigeration (Santos et al., 2021) or cryopreservation (Moreira et al., 2022) of its male germplasm. In 2020, the aerobic microbiota present in the semen and foreskin of these animals was reported to be primarily composed of several types of Gram-positive bacteria, and the use of antibiotics was therefore indicated for its control (Santos et al., 2020).

In the formulation of extenders for semen, not only for peccaries (Moreira et al., 2022), but also for countless species, the combination of penicillin and streptomycin (Almquist, 1951; Bryła and Trzcińska, 2015) and the gentamicin (Waberski et al., 2019) stand out as the most common antibiotics to guarantee the sanitary quality of the semen doses. In fact, these drugs can prevent bacterial proliferation, but they may also cause damage to sperm viability (Pinart et al., 2017; Bonet et al., 2018) according to its type and concentration (Aurich and Spergser, 2007). In this sense, determining the toxicity of these substances to sperm is of fundamental importance to ensure the quality of semen samples to be used both for the formation of biobanks or for the immediate use in other assisted reproductive technologies as in vitro fertilization or artificial insemination, so necessary for the conservation of valuable genotypes that will guarantee the survival of wild species.

Tests that lead the sperm to exhaustion when kept at temperatures close to body temperature, also called thermoresistance tests, make it possible to assess the ability of the sperm to survive and maintain fertility for long periods (Santos et al., 2011). In men, sperm motility has been considered the parameter most likely to be influenced by external agents, and therefore, its observation has been considered as a unique means to assess the toxicity of reagents and materials used in semen processing (Long et al., 2018). A multiparametric approach that evaluates multiple functional and morphological aspects, however, can provide more subsidies to verify the action of certain substances on the sperm cell as previously demonstrated when extenders for peccary semen preservation were evaluated (Campos et al., 2014). In this sense, a multiparametric thermoresistance test will allow the evaluation of sperm sensitivity to antibiotics, thus contributing to verify the safety of drugs added to the extender used to preserve collared peccary semen.

The present study aimed to evaluate the toxicity of the antimicrobial agents streptomycin-penicillin and gentamicin, added to the semen extender, on multiple morphological and functional parameters of collared peccaries sperm during a thermoresistance test at 37 °C for 180 min.

## Methods

The Animal Use Ethics Committee of Federal Rural University of Semiarid – UFERSA (No. 23091.009851/2018-96) and the Chico Mendes Institute for Biodiversity Conservation (No. 37329) approved this study. Reagents used were obtained from Sigma Chemical Co. (St. Louis, MO, USA) unless otherwise specified.

### Animals

The experiment was conducted with animals from the Center for Wild Animals Multiplication (CEMAS) of UFERSA, registered with IBAMA as a scientific breeding under number 1478912, located in Mossoró, Brazilian semi-arid region (5°10’S-37°10’W; average temperature range, 27-29 °C). Six sexually mature male (mean age 40 months) were used. The animals were exposed under natural outdoor photoperiod (~12 h) and segregated in groups of three in paddocks (20 m × 3 m) with covered area of 6 m^2^. They were fed with pig feed and fruits, and *ad libitum* access to water.

### Semen collection

The animals were fasted for 12 h prior to the semen collection procedure. They were restrained with a hand net and anesthetized through intravenous administration of propofol (Propovan®, Cristália, Fortaleza, Brazil) in bolus (5 mg/kg). The animals were monitored for their cardiac and respiratory parameters throughout the experimental procedures (Souza et al., 2009). Semen was collected using an electroejaculator (Autojac®, Neovet, Campinas, SP, Brazil), connected to a 12 V source, as previously stated for the species (Castelo et al., 2010). The stimulation cycle consisted of 10 stimuli at each voltage, starting at 5 V, followed by a 1 V increase to 12 V. Each electrical stimulus lasted 3 seconds, with intermittent intervals of 2 seconds. The stimulation cycle lasted 10 minutes. A rectal probe (15 cm long and 1.3 cm diameter) was inserted approximately 12 cm in the rectum. Ejaculates were collected in plastic tubes and immediately evaluated.

### Experimental design

The experiment was carried out in order to evaluate the existence of adverse effects of streptomycin-penicillin combination (Sigma, Sigma-Aldrich, Sao Paulo, SP, Brazil) and gentamicin (Gentatec®, Chemitec®, São Paulo, SP, Brazil) added to the Tris-citrate-fructose extender during the thermoresistance test at 37 °C of the collared peccaries’ fresh semen (Campos et al., 2014). Semen diluted in Tris extender alone was used as the control group; the test groups were composed of semen diluted in Tris plus streptomycin-penicillin (2 mg/ml-2000 IU/ml) or Tris plus gentamicin (70 µg/ml). Antimicrobial doses were used as previously reported in studies related to peccary semen cryopreservation (Moreira et al., 2022). All groups were adjusted to the same sperm concentration (100 x 10^6^ sperm/mL). Samples were stored in a water-bath at 37 °C for a thermoresistance test, in which they were evaluated for membrane integrity and functionality, mitochondrial activity, sperm morphology and kinetic parameters by means of computerized analysis. The assessments in the diluted samples were carried out at 0, 30, 60, 120 and 180 min.

### Semen evaluation

After collection, semen was immediately evaluated for volume, appearance, color and pH. To determine sperm concentration, a 10 µl semen aliquot was diluted in 2 mL buffered formaldehyde solution (10%) and analyzed in a Neubauer counting chamber (Silva et al., 2014).

For the analysis of membrane integrity and mitochondrial activity, a semen aliquot (10μL) was incubated at 37 °C for 10 min in a solution composed of the following combination of fluorescent probes: 2 μL Propidium Iodide (PI, Sigma-Aldrich, St. Louis, USA), 5 µl CMXRos (Mito Tracker® Red, Invitrogen®, Oregon, USA), and 3 μL Hoechst 342 (H342, Sigma-Aldrich, St. Louis, USA) (Souza et al., 2016). Next, the samples were evaluated with an epifluorescence microscope (Episcopic Fluorescent Attachment “EFA” Halogen Lamp Set, Leica, Kista, Sweden), and 200 spermatozoa (per sample) were evaluated for the plasma membrane integrity using PI/H342 association and for mitochondrial membrane potential through CMXRos. Sperm heads marked in blue (H342) were considered to have intact membrane and those totally or partially marked with red (PI) were considered to be not intact; region of the midpiece marked in red was considered as presenting mitochondrial activity (Souza et al., 2016)

The hypo-osmotic test was performed to assess sperm osmotic response, which is related to the sperm membrane functionality. The assay was conducted by using distilled water (0 mOsm/l) as a hypo-osmotic solution. A total of 200 cells were counted in which those with curly tail were judged as having a functional membrane under light phase contrast microscopy (Alttion®, Wuzhou City, Guangxi Province, China) (Santos et al., 2013).

For sperm morphological analysis, semen smears were stained with Bengal Rose (Sigma-Aldrich, St. Louis, USA), being observed 200 cells/slide under light microscopy (×1000). Sperm morphology was classified according to head, midpiece and tail defects (Sousa et al., 2013).

Sperm motility parameters were evaluated by an automated IVOS 7.4G system (Hamilton-ResearchTM Thorne, Beverly, MA, USA) in fresh semen using the settings previously stablished for the species (Souza et al., 2016). The following parameters were evaluated: total motility (%), velocity average pathway (VAP, µm/s), velocity straight line (VSL, µm/s), velocity curvilinear (VCL, µm/s), amplitude lateral head (ALH, µm), beat cross frequency (BCF, Hz), straightness (STR, %), and linearity (LIN, %) as well as the sperm subpopulations: rapid, medium, slow and static.

### Statistical analysis

We used a total of six samples in the study, being one ejaculate per individual. Sperm characteristics were expressed as Mean ± SE. The data were first examined for normality by the Shapiro–Wilk test and for homoscedasticity by Levene’s test and were transformed by log (x + 1) or arcsine (√ (x/100)), when necessary. A two-way ANOVA using a general linear model using the PROC GLM procedure of the Statistical Analysis System (SAS Institute Inc.) was performed to evaluate the effects of the treatment, incubation time (0, 30, 60, 120 and 180 min) and its interaction on the studied parameters. Tukey post hoc test was used to verify the potential differences between the means. Statistical significance was set at P < 0.05.

## Results

### Fresh semen characteristics

The assessment of fresh semen presented a watery aspect, white color, and a volume of 1.8 ± 0.7 mL with a concentration of 503.3 ± 112.6 x 10^6^ sperm/mL. Average values of 78.8 ± 3.9% motile sperm, being 86 ± 4.1% with membrane integrity, 87.2 ± 3.7% with mitochondrial activity, 78.5 ± 3.5% morphologically normal, and 65.5 ± 6.2% with membrane functional were found in ejaculates samples.

### Effect of antibiotics on sperm morphologic and functional parameters

**Figure 1:**
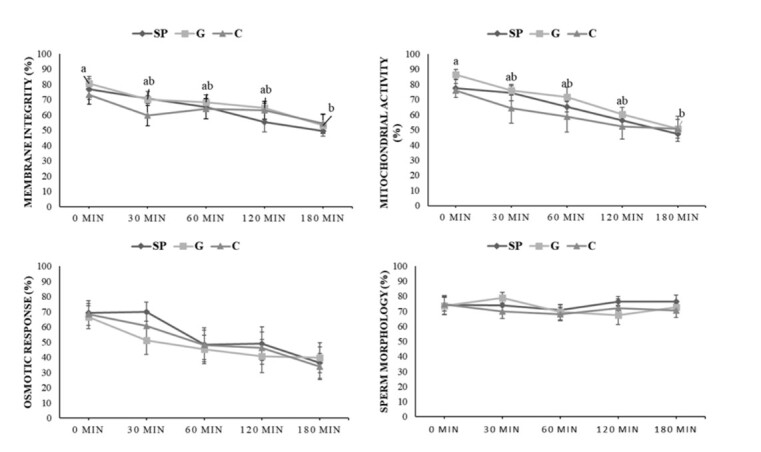
Values (mean) for membrane integrity (%), mitochondrial activity (%), osmotic response (%) and sperm morphology (%) on collared peccary (*Pecari tajacu*) fresh semen samples diluted in Tris-citrate-frutose without (C) and with streptomycin-penicillin (SP) or gentamicin (G) up to 180 min (n = 6). (a-b) lowercase letters indicate significant differences for treatments on time (P < 0.05).

During thermoresistance test for 180 min, there were no differences (P > 0.05) among the treatments diluted with streptomycin-penicillin and gentamicin compared to the control (Tris alone) for integrity and functionality of the plasma membrane, mitochondrial activity and sperm morphology (Figure 1). When assessing the progression of the variables for each treatment along the time, however, it was observed that the streptomycin-penicillin combination did not differ (P > 0.05) from the control, with both keeping the mean values stable up to 180 min (with total motility of 24.3 ± 7.1% and 28 ± 8.7% at this time, respectively). On the other hand, gentamicin provoked a reduction (P < 0.05) at 180 min, compared to 0 min, of membrane integrity (80.5 ± 4.7% at 0 min to 53.1 ± 7.1% 180 min) and mitochondrial activity (86.3 ± 3.4% at 0 min to 50.7 ± 6.2% at 180 min) of the sperm.

Regarding the analysis of kinetic parameters as assessed by CASA, the data are shown in Table1 and Table 2. No differences were observed (P > 0.05) among treatments or different incubation times for the kinetic variables and for the sperm subpopulations evaluated up to 180 min.

**Table 1:** Values (mean ± SEM) for total (TM, %) and progressive (PM, %) motility, average path velocity (VAP, µm/s), straight line velocity (VSL, µm/s), curvilinear velocity (VCL, µm/s), amplitude of lateral head (ALH, µm), beat cross frequency (BCF, Hz), straightness (STR, %), linearity (LIN, %) on collared peccary (*Pecari tajacu*) fresh semen samples diluted in Tris-citrate-frutose without (control) and with streptomycin-penicillin (SP) or gentamicin (G) up to 180 min (n = 6).

**Treatments**	**Time (min)**	**Kinetics parameters**								
		**TM**	**PM**	**VAP**	**VSL**	**VCL**	**ALH**	**BCF**	**STR**	**LIN**
Tris-control	0	66.3 ± 9.7	31.3 ± 8.6	38.6 ± 3	23.5 ± 2	80.4 ± 7.3	5.9 ± 0.3	29.2 ± 1.6	59.8 ± 2.2	30.3 ± 1.3
	30	48.8 ± 12.8	21.3 ± 6.6	44.8 ± 4.5	26.1 ± 2.5	86.5 ± 8.1	6 ± 0.3	28.7 ± 1.6	53.3 ± 1	29.2 ± 1.4
	60	47.3 ±7.2	17.1 ± 4.7	39.2 ± 2.3	20 ± 1.2	78.3 ± 5.7	5.6 ± 0.2	29.2 ± 1.6	49.7 ± 1.1	26 ± 0.4
	120	37 ± 9.7	13.8 ± 3.9	37.7 ± 3.8	20.5 ± 2.4	75.5 ± 5.6	5.6 ± 0.3	26.9 ± 1.3	52.3 ± 3.3	26 ± 1.5
	180	24.3 ± 7.1	5.1 ± 3	32.9 ± 5.8	14.2 ± 1.3	66.7 ± 12	4.9 ± 0.5	28.2 ± 1.5	48.5 ± 2.1	26.1 ± 2.1
Tris-SP	0	57.2 ± 12.2	30 ± 10.4	39.7 ± 3.6	24.4 ± 2.4	86 ± 7.9	6 ± 0.3	30.4 ± 1.6	58.7 ± 3.5	28.2 ± 1.2
	30	53.8 ± 13	26.2 ± 10.3	40 ± 5.4	23 ± 3.4	85.8 ± 10.3	6.3 ± 0.4	30.8 ± 2	55.8 ± 2.1	27 ± 0.7
	60	50.8 ± 14.4	25.5 ± 8.8	37.2 ± 5.6	23 ± 2.3	82.3 ± 11.2	6 ± 0.4	25.9 ± 2.6	57.3 ± 1.2	29 ± 2
	120	56.7 ± 10.4	25 ± 7.2	34.2 ± 3.1	19.6 ± 1.8	78.3 ± 6	5.9 ± 0.3	29.4 ± 2	55.2 ± 1.3	25.2 ± 0.6
	180	28 ± 8.7	8.7 ± 4.6	32 ± 2.8	17.7 ± 1.6	72.1 ± 4	5.2 ± 0.4	28.1 ± 1.7	52.5 ± 1.1	24.8 ± 1.3
Tris-G	0	52.5 ± 10.9	25.7 ± 10	38.4 ± 2.7	22.9 ± 2.1	82.8 ± 6.9	5.8 ± 0.2	29.5 ± 1.2	57.8 ± 3.3	28 ± 1.5
	30	52 ± 15.2	29.1 ± 10.2	41.4 ± 4.1	24.8 ± 2	87.8 ± 8.5	5.8 ± 0.3	26.3 ± 0.9	59.5 ± 2.4	29.2 ± 0.7
	60	47.3 ± 13.6	19.5 ± 6.8	37.1 ± 2.6	21.3 ± 1.6	81.9 ± 8.5	5.9 ± 0.2	28.5 ± 2.5	56.1 ± 3.1	25.8 ± 1.7
	120	21 ± 5.5	4.5 ± 1.5	30.3 ± 2.8	15.2 ± 0.5	68.9 ± 4.8	5.2 ± 0.3	29.4 ± 2.4	50 ± 3.4	22.8 ± 1.7
	180	18.5 ± 3.1	2 ± 0.6	24.5 ± 2.1	13.3 ± 1.3	57.2 ± 3.5	4.5 ± 0.5	33.2 ± 2.4	55.3 ± 2.5	26 ± 1.6

No differences were found between treatments and storage times (P > 0.05).

**Table 2:** Values (mean ± SEM) for motile subpopulations on collared peccary (*Pecari tajacu*) fresh semen samples diluted in Tris-citrate-frutose without (control) and with streptomycinpenicillin (SP) or gentamicin (G) up to 180 min (n = 6).

**Treatments**	**Time (min)**	**Motile subpopulations**			
		**Rapid (%)**	**Medium (%)**	**Slow (%)**	**Static (%)**
Tris-control	0	44.8 ± 6.6	0.5 ± 4.7	0.2 ± 1.1	28.7 ± 9.9
	30	36.5 ± 10.3	0.3 ± 3.6	0.3 ± 0.9	44.5 ± 12.6
	60	33.8 ± 6.7	0.4 ± 3	0.3 ± 0.9	44.8 ± 6.8
	120	28 ± 8.6	0.3 ± 3	0.2 ± 1.2	57.3 ± 10.8
	180	11.8 ± 6.2	0.3 ± 4.1	0.2 ± 0.6	70.8 ± 7.6
Tris-SP	0	40.3 ± 4.4	0.4 ± 4.4	0.2 ± 1	38 ± 12
	30	39.5 ± 3.2	0.4 ± 3.2	0.2 ± 2	40.2 ± 12.8
	60	37.7 ± 3.4	0.3 ± 3.4	0.2 ± 0.9	44.8 ± 14.8
	120	37.8 ± 2.7	0.4 ± 2.7	0.2 ± 1.1	37.3 ± 10.2
	180	16.5 ± 3.2	0.3 ± 3.2	0.2 ± 0.7	67.5 ± 8.8
Tris-G	0	35.8 ± 3.5	0.4 ± 3.5	0.2±0.9	42.3±10.8
	30	38.8 ± 4.5	0.3 ± 4.5	0.2±1.6	44±15.1
	60	31.8 ± 5	0.4 ± 5	0.2 ± 1.5	46.7 ± 14
	120	11.1 ± 3.2	0.3 ± 3.2	0.2 ± 1.1	75.5 ± 6.2
	180	5.5 ± 2.3	0.4 ± 2.3	0.2 ± 0.7	76.2 ± 3.4

No differences were found between treatments and storage times (P > 0.05).

## Discussion

Studies related to reproductive microbiomes, either regarding their composition or their control, are scarce in wild species (Santos et al., 2023). In this sense, the present study adds information to this subject since it proves the relative safety of the use of antimicrobial agents in semen extenders regarding potential effects on peccary sperm quality. This information then guarantees the safety of the tested antimicrobials, especially streptomycin-penicillin combination, as they do not greatly interfere with the potential of semen samples from collared peccaries to be used in *in vitro* fertilization (Silva et al., 2023) or artificial insemination protocols (Peixoto et al., 2019).

In a previous experiment on the sensitivity of bacterial strains isolated from collared peccary semen, our group demonstrated that concentrations of streptomycin-penicillin ranging from 0.5 mg/mL - 500 IU/mL to 2 mg/mL - 2,000 IU/mL are capable of to inhibit from 70% to more than 80% of the isolates, respectively. Continuing, in the present study, we found that the streptomycin-penicillin combination at a concentration of 2 mg/mL-2,000 IU/mL did not cause adverse effects on the structure and function of the sperm cell during the resistance test at 37 °C for 180 min. It is worth noting that this concentration used is twice that most used in semen extenders of domestic species such as bulls (Almquist, 1951), dogs (Lopes et al., 2009), rams (Moustacas et al., 2010) and buffaloes (Akhter et al., 2008). However, there is variation in doses for different species, from 38 µg/mL to 105 µg/mL (accompanied by 0.315 µg/mL amphotericin) in stallions (Dean et al., 2012) to 10 mg/ml10,000 IU/mL in wild canids (*Canis lupus* and *Canis lupus baileyi*) (Zindl et al., 2006).

The inclusion of streptomycin-penicillin and gentamicin alone in extenders is important in controlling or eliminating certain pathogens that may be present in semen (Moustacas et al., 2010), for example. Being able to guarantee the sanitary quality of the semen doses controlling possible contaminations during its handling (Moreira et al., 2022). Streptomycin and gentamicin are aminoglycoside antibiotics that block protein production by binding to the 30S ribosome, thereby inhibiting messenger RNA in the bacterial cell (Luzzatto et al., 1968; Hahn and Sarre, 1969), and penicillin is a β antibiotic. -lactam that interferes with bacterial cell wall synthesis, causing cell lysis and death (Waxman and Strominger, 1983).

In turn, gentamicin at a concentration of 70 µg/mL caused effects on plasma membrane integrity and mitochondrial activity of collared peccary semen after 120 min of incubation at 37 °C. Membrane integrity is essential for sperm function and survival, and loss of integrity can decrease fertilization capacity and lead to cell death (Rodriguez-Martinez and Barth, 2007). In addition, mitochondria produce ATP, which is an essential source of energy for sperm motility (Silva and Gadella, 2006). Despite this information, this antibiotic is usually used at higher doses, such as 250 mg/mL in pig semen extenders (Waberski et al., 2019) and is mainly used during the cooling of the semen of these animals (Bryła and Trzcińska, 2015). Likewise in peccaries, gentamicin has also been used for both refrigeration (Santos et al., 2021) and freezing (Moreira et al., 2022) of semen, and deleterious effects on the sperm cell have not been identified at such temperatures, emphasizing that its deleterious effect may be related to the temperature at which the drug is used.

It is also worth mentioning that some authors report that gentamicin can cause mitochondrial dysfunction and overproduction of reactive oxygen species (ROS) in mammalian cells, which lead to oxidative damage to DNA, membrane proteins and lipids (Kalghatgi et al., 2013). Mitochondria share ribosomes and protein synthesis machinery similar to bacteria (Elliott and Jiang, 2019). Gentamicin is also known to kill bacteria by binding to their 30S ribosomal unit and thereby affecting protein synthesis (Hahn and Sarre, 1969). Thus, it is suggested that the mitochondria of eukaryotic cells, similar to evolutionary bacteria, would be affected by antibiotics in the same way as bacteria (Elliott and Jiang, 2019), suggesting a possible sperm toxic mechanism, as demonstrated in the present study with the collared peccary semen incubated at 37 °C. Among other effects caused by gentamicin in cultured mammalian cells, we can highlight the increase in the gene expression of the hypoxia inducing factor 1 alpha (HIF1a), glycolytic enzymes and glucose transport in cultured mammalian cells, in addition to increasing the production of lactate from cell lines (Elliott and Jiang, 2019).

It is also worth noting that maintaining the viability of the semen sample at 37 °C for a long period helps to guarantee the efficiency of the procedure, especially when working with wild animals, given the possibility of complications during the process. A great example can be seen when carrying out artificial insemination in the species, in which fresh semen could remain for periods of up to 30 minutes before being deposited in the female's genital tract (Peixoto et al., 2019). Of course, we need to recognize that the work may present some limitations, mainly due to the intrinsic difficulties of managing a wild species, which often limits the experimental number to be used. However, given the nature of this difficulty, the results are valid and applicable, especially at a time when the adoption of an assisted reproduction program aimed at conserving both peccary and related species is envisaged.

## Conclusions

In conclusion, the thermoresistance test proved the safety of using the combination streptomycin-penicillin on the morphological and functional parameters of spermatozoa from peccaries. On the other hand, gentamicin, when at 37 °C, seems to have a deleterious effect mainly on the plasmatic membrane and sperm mitochondrial activity in this species.
